# Anti-Inflammatory Effects of Olive Leaf Extract and Its Bioactive Compounds Oleacin and Oleuropein-Aglycone on Senescent Endothelial and Small Airway Epithelial Cells

**DOI:** 10.3390/antiox12081509

**Published:** 2023-07-28

**Authors:** Andrea Silvestrini, Chiara Giordani, Sonia Bonacci, Angelica Giuliani, Deborah Ramini, Giulia Matacchione, Jacopo Sabbatinelli, Silvia Di Valerio, Deborah Pacetti, Antonio Domenico Procopio, Antonio Procopio, Maria Rita Rippo

**Affiliations:** 1 Department of Clinical and Molecular Sciences, DISCLIMO, Università Politecnica delle Marche, 60126 Ancona, Italyj.sabbatinelli@staff.univpm.it (J.S.);; 2 Department of Health Sciences, University Magna Graecia of Catanzaro, 88100 Catanzaro, Italy; 3 Clinic of Laboratory and Precision Medicine, IRCCS—National Institute for the Care of the Elderly (INRCA), 60121 Ancona, Italy; debby.ramini@gmail.com; 4 Department of Agricultural, Food and Environmental Sciences, Marche Polytechnic University, Via Brecce Bianche, 60131 Ancona, Italy

**Keywords:** olive leaf extract, oleacin, oleuropein-aglycone, antioxidant, anti-inflammatory, senescence, respiratory infections

## Abstract

Olive tree by-products have been deeply studied as an invaluable source of bioactive compounds. Several in vitro and in vivo studies showed that olive leaf extract (OLE) has anti-inflammatory and antioxidant properties. Here, we wanted to assess the valuable benefits of two less-studied OLE components—3,4-DHPEA-EDA (Oleacin, OC) and 3,4-DHPEA-EA (Oleuropein-Aglycone, OA)—directly purified from OLE using a cost-effective and environmentally sustainable method, in line with the principles of circular economy. OLE, OC and OA were then tested in human cellular models involved in acute and chronic inflammation and in the pathogenesis of viral infections, i.e., lipopolysaccharide (LPS)-treated monocyte/macrophages (THP-1) and endothelial cells (HUVECs), senescent HUVECs and Poly(I:C)-treated small airway epithelial cells (hSAECs). Results showed that OC and OA are efficient in ameliorating almost all of the pro-inflammatory readouts (IL-1β, TNF-α, IL-8, ICAM, VCAM) and reducing the release of IL-6 in all the cellular models. In hSAECs, they also modulate the expression of SOD2, NF-kB and also ACE2 and TMPRSS2, whose expression is required for SARS-CoV-2 virus entry. Overall, these data suggest the usefulness of OLE, OC and OA in controlling or preventing inflammatory responses, in particular those associated with viral respiratory infections and aging.

## 1. Introduction

The human and animal health effects of chemical compounds that can be found in the fruit and leaf of *Olea europaea* L. tree have been known for a long time, especially those of oleuropein and hydroxytyrosol [1,2,3]. Olive leaf extract (OLE) showed similar properties to extra-virgin olive oil (EVOO), one of the main components of the Mediterranean Diet, whose qualities have long been studied [4,5]. Many in vitro and in vivo reports throughout the years demonstrated that OLE and EVOO reduce oxidative stress and inflammation [6,7,8], but their valuable effects were also found in cardiovascular diseases [9,10,11], metabolic disorders [12,13,14] and bacterial infections [15,16].

The main constituents of OLE are polyphenols; they are produced in leaves as protective molecules (phytoalexins) against leaf-eating insects, microbes and fungi [17,18]. Polyphenol total concentration in *Olea europea* L. tree decreases during summer, progressively increases in autumn and reaches its higher level at the beginning of winter. Therefore, olive leaves can be valorised more efficiently as olive by-products during EVOO production (defoliation of olives delivered to the mill) or during pruning [19].

Oleuropein, an ester of elenolic acid and 3,4-dihydroxyphenyl ethanol belonging to the secoiridoids family, is the major phenolic component found in olive leaves followed by other secoiridoids derived from 2-(4-hydroxyphenyl) ethanol (tyrosol). Oleuropein content depends upon the cultivar, ripening stage and extraction methods [20,21]. Notably, oleuropein concentration in olives is higher as compared to leaves but it is hydrolysed during olive oil production [22]. Therefore, the beneficial properties of EVOO are due to the oleuropein degradation products: its aglycone 3,4-DHPEA-EA (oleuropein aglycone) and dialdehydic forms 3,4-DHPEA-EDA (oleacein) and 3,4-DHPEA (hydroxytyrosol) [23]. However, there are many difficulties in isolating oleacein and oleuropein aglycone as pure compounds from olives, not to mention the high-priced extraction process.

Polyphenols have long been studied as antioxidant and anti-inflammatory agents; in the last decade, their efficacy has been demonstrated in the reduction of inflammaging. [24,25,26]. Inflammaging is a subclinical systemic inflammation observed during the aging process. Aging also entails an acquired immune system impairment (immune senescence). The main culprit of both inflammaging and immune senescence is the acquisition of a senescence-associated secretory phenotype (SASP) by senescent cells [27]. The main feature of SASP is the release of high levels of pro-inflammatory cytokines [24]. Altogether, these conditions contribute to the development of age-related diseases.

The experience and research following the COVID-19 pandemic showed that in elderly men with age-related comorbidities, viral infections could cause high mortality. In most of these patients, SARS-CoV-2 induces an uncontrolled local and systemic hyperinflammation (cytokine storm) with serious harmful acute respiratory distress syndrome and local bacterial/fungal superinfections, due to impaired microbicide capacity [28] and multi-organ failure [29,30,31]. In viral infections, this hyperinflammation is also promoted by the imbalance of redox homeostasis. This phenomenon has also been demonstrated for SARS-CoV-2 which spoils the redox homeostasis in the airways, leading to an increased replication and the entrance of SARS-CoV-2 into host cells [32].

Timely targeted strategies based on antioxidant drugs and viral-induced cytokine inhibitors would certainly improve the clinical outcome of infectious diseases, also in elderly people.

In light of the above-mentioned issues to obtain sufficient amounts of pure compounds from olive leaves, their beneficial effect in chronic inflammation associated with aging and acute inflammation associated with viral infection has still been poorly explored. Of note, our research group has developed a new environmentally friendly extraction method to obtain pure bioactive compounds from olive leaves, in line with circular economy principles and the 2030 Agenda for Sustainable Development [24,31,32,33].

Therefore, here, we wanted to explore the anti-inflammatory properties of (i) a total olive leaf aqueous extract (OLE) and (ii) its less-studied derivatives oleuropein aglycone and oleacein, both produced in our laboratory, in in vitro human models of acute and chronic inflammation. For this purpose, we used (i) human LPS-treated primary vascular endothelial cells (HUVECs) and the monocytic cell line THP-1, (ii) replicative senescent HUVECs (RS-HUVECs)—the most studied and representative of SASP—and (iii) Poly(I:C)-treated primary small airway epithelial cells (hSAECs) as a model to study the molecular mechanisms underlying respiratory viral infections.

## 2. Materials and Methods

### 2.1. Sample Collection and Preparation

**Figure 1:**
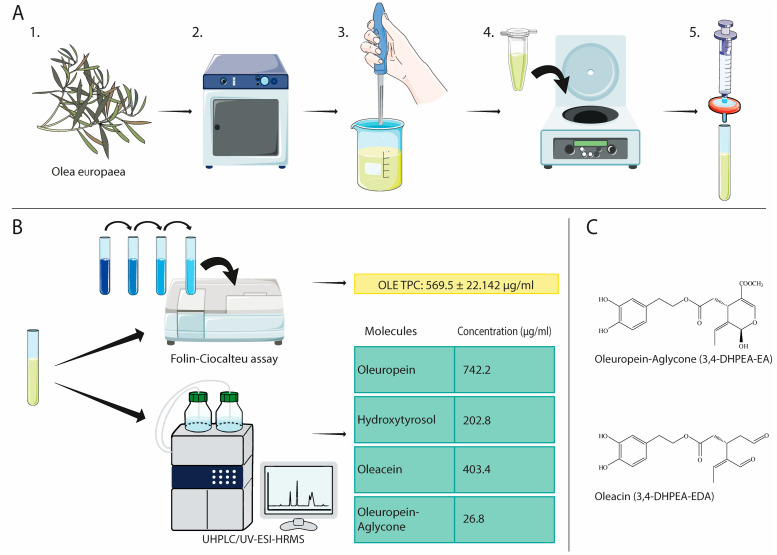
Olive leaf extract preparation steps and characterisation. (**A**) Freshly harvested olive leaves (1) were freeze-dried and vacuum-sealed using a lyophiliser (2); upon use, olive leaves were ground with a homogeniser to obtain an uneven powder (3) which was immediately used for TPC extraction in PBS; the extract was centrifuged (4), the supernatant filtered (5) and finally stored at −20 °C before downstream applications. (**B**) OLE’s TPC was determined using the Folin–Ciocalteau reagent, whereas OLE’s main compounds were determined via UHPLC/UV-ESI-HRMS. (**C**) Molecular structure of OA and OC. This figure was created using the Servier Medical Art Commons Attribution 3.0 Unported Licence (http://smart.servier.com, accessed on 5 July 2023).

Olive leaves were collected in November from cultivar Leccino in the Marche region (Italy). Trees were grown without any kind of chemical treatment. Samples were washed with distilled water, dried and lyophilised using a benchtop freeze drier (Virtis Wizard 2.0 instrument, SP Industries, New York, NY, USA). Olive leaves were vacuum-sealed and stored at room temperature in the dark. Upon use, olive leaves were ground with a homogeniser to obtain an uneven powder which was immediately used for extraction (Figure 1).

### 2.2. Olive Leave Extraction Procedure

An amount of 5. gr of olive leaf powder was macerated in 50 mL PBS at 4 °C for 24 h in the dark to maximise the extraction and avoid degradation. The resulting extract was centrifuged at 1500 RPM for 10 min to separate coarse particles and then filtered through a 0.22 μm membrane filter to ensure sterility. The extract was aliquoted and stored at −20 °C until use for cell treatments.

### 2.3. OLE Composition

The Folin–Ciocalteu assay, with minor modifications, was used to determine OLE’s total phenolic content (TPC). Different concentrations of Gallic acid were used as standard. An amount of 1 mL of Folin–Ciocalteau reagent diluted 1:10 in distilled water was mixed with 0.1 mL of OLE or standard. An amount of 0.9 mL of Na_2_CO_3_ solution (7.5% *w*/*v*) was added precisely after 3 min and incubated at room temperature for 90 min, protected from light. The TPC amount was determined by measuring the optical density (OD) at 760 nm using a microplate reader (MPT Reader, Invitrogen, Milano, Italy).

### 2.4. UHPLC-UV-ESI-HRMS Analysis

The chemical characterisation of OLE main compounds was carried out at the Department of Health Sciences of the University Magna Graecia of Catanzaro. UHPLC/UV-ESI-HRMS analyses for quantification of the extract were performed by reverse-phase ultra-high-performance liquid chromatography followed by high-resolution mass spectrometry, with ionisation in negative mode. Chromatography separation was performed using a Dionex Ultimate 3000 RS (Thermo Scientific—Rodano, MI, Italy), equipped with a Hypersil Gold C18 column (100 × 2.1 mm, 1.9 µm particle size, Thermo Scientific) and a mobile phase consisting of the following solvents: 98% A (H_2_O + 0.1% formic acid) and 2% B (methanol). The UV/VIS detector was set at 235, 254, 280 and 330 nm. Mass detection was performed by a high-resolution Q-Exactive orbitrap mass spectrometer (Thermo Scientific, Rodano, MI, Italy). Heated electrospray ionisation (HESI) was selected in negative polarity, with the following operating conditions: 70,000 resolving power (defined as FWHM at *m*/*z* 200), IT 100 ms, ACG target = 1 × 10^6^ and scan range (100–900 *m*/*z*). In each scan, the negative exact mass [M-H]− of biophenols precursors was selected (Hydroxytyrosol 153.0557 *m*/*z*; Oleacein 319.1187 *m*/*z*; Oleuropein Aglycone 377.1248 *m*/*z*; Oleuropein 539.1770 *m*/*z*) in Parallel Reaction Monitoring (PRM). MS/MS analyses were performed according to the following operating conditions: resolution: 35.000; AGC target = 1 × 10^5^; maximum IT 200 ms; collision energy (stepped NCE): 20,30,40. The quadrupole isolation window was set to 2.0 *m*/*z*. High-purity nitrogen was used as the sheath gas (30 arb units) and auxiliary gas (10 arb units). Xcalibur software (version 4.1, Thermo Fisher Scientific) was used for instrument control, data acquisition and data analysis.

### 2.5. Preparation of 3,4-DHPEA-EA (Oleuropein Aglycone) and 3,4-DHPEA-EDA (Oleacein)

Oleuropein aglycone (3,4-DHPEA-EA) was obtained from the controlled hydrolysis of oleuropein extracted from olive leaves as previously described [33,34]. Briefly, oleuropein (1.0 mM) dissolved in aqueous CH_3_CN (10 mL) was refluxed for 8 h at 80 °C in the presence of 10 mol% of Er(OTf)_3_. The hydrolysate was cooled, 5 mL of water was added and the mixture was extracted with CH_2_C_l2_. After drying on Na_2_SO_4_, the organic solvent was removed in vacuo and the crude product was purified by flash chromatography (total yield = 70%).

Oleacein (3,4-DHPEA-EDA) was obtained via one step semi-synthesis under microwave-assisted aqueous Krapcho decarbomethoxylation of OLE extracted from the olive leaves of *Olea europaea* L., cultivar Coratina, as previously described [23]. Briefly, a water solution of oleuropein (0.1 mM in 0.5 mL of water) was put into a 3.0 mL glass vial, NaCl (2.0 equivalents) was added, it was sealed and then reacted in a Synthos 3000 Microwave oven by Anton Paar. At the end of the reaction (20 min), the oleuropein conversion was complete. The reaction crude was recovered by adding 0.5 mL of EtOH and purified by flash column chromatography to give pure oleacein (48% total yield).

### 2.6. Cell Culture

Primary Human Umbilical Vein Endothelial Cells (HUVEC) were obtained from a pool of donors and purchased from Clonetics (Lonza, Basel, Switzerland). Cells were maintained in a humidified atmosphere at 37 °C and 5% CO_2_, seeded at a density of 5000/cm^2^ in T75 flasks (Corning Costar, Sigma Aldrich, St. Louis, MO, USA), with endothelial growth medium (EGM-2), composed of endothelial basal medium (EBM) and the SingleQuots Bullet kit (Lonza, Switzerland). The medium was changed every 48 h and cells were trypsinised when approximately 80% confluent.

Replicative senescence was achieved after several replicative passages (measured as cumulative population doubling, cPD). cPD was calculated as the sum of PD changes, using the formula (log10 (F) − log10 (I))/log10, where F is the number of cells at the end of a passage, and I is the number of seeded cells. HUVECs were classified as young or senescent based on cPD, senescence-associated (SA)-β-Galactosidase activity and p16ink4a expression as previously described by our research group [24].

Human monocytic THP-1 cells were purchased from ATCC (Rockville, MD, USA) and grown in RPMI-1640 culture medium supplemented with 2-mercaptoethanol to a final concentration of 0.05 mM and with 10% heat-inactivated foetal bovine serum, 1% penicillin/streptomycin and 1% L-glutamine (all from Euroclone, Milano, Italy) at 37 °C in 5% CO_2_ in a humidified incubator. The cells were seeded at a density of 2 × 10^5^ cells/ml in T75 flasks.

Primary human small airway epithelial cells (hSAEC) were purchased from ATCC (PCS-301-010) and grown to confluence in a humidified atmosphere at 37 °C and 5% CO_2_, seeded at a density of 5000/cm^2^ with Airway Epithelial Cell Basal Medium (ATCC. PCS-300-030) and the bronchial epithelial cells growth kit (ATCC, PCS-030-040). The medium was changed every 48 h and cells were trypsinised when approximately 80% confluent.

All cell lines have been tested for Mycoplasma.

### 2.7. Cell Viability Assay

Cell viability was assessed through the MTT (3-(4,5-dimethylthiazol-2-yl)-2,5-diphenyltetrazolium bromide) assay. Cells were grown for 24 h in 12-well plates at a density of 5000/cm^2^ (HUVEC and hSAEC) or 2 × 10^5^ cells/mL (THP-1) and then treated with different amounts of OLE (different doses in terms of TPC) or with different concentrations of Oleacein and Oleorupein-Aglycone for 24 h. As concerns Oleacin, the concentration range tested was 1–100 μM (0.32–32 μg/mL), whereas Oleuropein-Aglycone was 1–100 μM (0.36–36 μg/mL). Untreated cells were used as the control group. MTT solution (5 mg/mL) was added in each well (10 µL/100 µL Medium) and incubated for 2.5 h. Insoluble formazan salts produced were solubilised by adding DMSO (400 μL). The absorbance was measured at OD 540 nm using a microplate reader (MPT Reader, Invitrogen, Milano, Italy). Data are expressed as a percentage, according to the equation (T/C) × 100%, where T and C represent the mean OD of treated cells and untreated cells (control group), respectively.

### 2.8. Cell Treatments

Based on the results of the viability assay, young HUVECs were seeded at a density of 5000/cm^2^; after 24 h, cells were pre-treated with OLE (4 μg/mL) or with Oleacein (5 μM) or Oleorupein-Aglycone (5 μM) for 2 h and then stimulated with lipopolysaccharide (LPS) (500 ng/mL) for three hours to induce an inflammatory response. Untreated cells were used as a control to ensure the inflammatory effect of LPS. THP-1 cells were seeded at a concentration of 2 × 10^5^/mL in suspension and treated with the same conditions after 24 h. RS-HUVECs were seeded at a density of 5000/cm^2^ and treated with OLE or with the single compounds for 24 h to evaluate their effect on the basal inflammatory status. hSAEC cells were seeded at a density of 5000/cm^2^ and pre-treated with OLE (4 μg/mL) or with the single compounds (1 μM OC, 5 μM OA) for 2 h, followed by a 24 h treatment with Poly(I:C) (5 μg/mL), according to the literature [35,36].

### 2.9. RNA Isolation and mRNA Expression by RT-qPCR

Total RNA was isolated using the Total RNA Purification kit (Norgen Biotek Corp., #37500, Thorold, ON, Canada), following the manufacturer’s instructions and stored at −80 °C until use. Total RNA quantification was determined by spectrophotometric quantification with Nanodrop ONE (NanoDrop Technologies, Wilmington, DE, USA) and reverse transcribed using PrimeScriptTM RT Reagent Kit with gDNA eraser (Cat. #RR037B, Takara), following manufacturer’s instructions. mRNA expression was evaluated by RT-qPCR using TB GreenTM Premix Ex TaqTM (Cat#RR420A, Takara) in a Rotor-Gene Q (Qiagen). The following primers were all acquired from Merck Millipore (Darmstadt, Germany): β-actin (FW: 5′-AACTGGAACGGTGGTCAAGGTGAC-3′, RV: 5′CAAGGGACTTCCTGTAACAATGC-3′), IL-1β(FW: 5′-AGATGATAAGCCCACTCTACAG-3′, RV: 5′-ACATTCAGCACAGGACTCTC-3′), IL-6 (FW: 5′-TGCAATAACCACCCCTGACC-3′, RV: 5′-GTGCCCATGCTACATTTGCC-3′), IL-8 (FW: 5′-GGACAAGAGCCAGGAAGAAA-3′, RV: 5′-CCTACAACAGACCCACACAATA-3′), NF-kB (FW: 5′-ACAGCTGGATGTGTGACTGG-3′, RV: 5′-TCCTCCGAAGCTGGACAAAC-3′), TNF-α (FW: 5′-AAGCCTGTAGCCCACGTCGTA-3′, RV: 5′-GGCACCACTAGTTGGTGGTCTTTG-3′), ICAM1 (FW: 5′-AGCCAGGAGACACTGCAGACA-3′, RV: 5′-TGGCTTCGTCAGAATCACGTT-3′), VCAM (FW: 5′-GGGAAGATGGTCGTGATCCTT-3′, RV: 5′-TCTGGGGTGGTCTCGATTTTA-3′), ACE2 (FW: 5′-GGGATCAGAGATCGGAAGAAGAAA-3′, RV: 5′-AGGAGGTCTGAACATCATCAGTG-3′), TMPRSS2 (FW: 5′-ACTCTGGAAGTTCATGGGCAG-3′, RV: 5′-TGAAGTTTGGTCCGTAGAGGC-3′), SOD2 (FW: 5′-GTTGGGGTTGGCTTGGTTTC-3′, RV: 5′-ATAAGGCCTGTTGTTCCTTGC-3′). mRNA quantification was assessed using the 2^−ΔΔCt^ method. β-actin was used as endogenous control.

### 2.10. Western Blot

Cell lysates were obtained using RIPA buffer (10 mM Tris, pH 7.2, 150 mM NaCl, 1.0% Triton X-100, 0.1% SDS, 5 mM EDTA, pH 8.0) with a protease inhibitor cocktail (Roche Applied Science, Indianapolis, IN, USA). Bradford assay was used to evaluate protein concentration in each sample. Proteins (25 μg) were analysed with SDS-PAGE and transferred to a nitrocellulose membrane (Bio-Rad, Hercules, CA, USA). Then, 5% skim milk was used to block the membrane, which was then incubated overnight with the primary antibody. Rabbit anti-phospho-NF-κB (Cell Signaling Technology, Danvers, MA, USA), rabbit anti-ACE2 (Cell Signaling), mouse anti-TMPRSS2 (Santa Cruz Biotechnology, Dallas, TX, USA), mouse anti-β-actin (Santa Cruz Biotechnology, Dallas, TX, USA) and rabbit anti-GAPDH (Cell Signaling Technology, Danvers, MA, USA) were used as primary antibodies. Horseradish peroxidase-conjugated antibodies anti-mouse or anti-rabbit (The Jackson Laboratory, Bar Harbor, ME, USA) were used as secondary antibodies. Protein bands were visualised by using the Clarity ECL chemiluminescence substrate (Bio-Rad) with Uvitec Imager (UVItec, Cambridge, UK) and then quantified using ImageJ software 4.1.

### 2.11. ELISA

HUVEC and hSAEC supernatants were collected after each treatment, centrifuged and stored at −80 °C until use. Interleukin-6 (human) ELISA Kit (Invitrogen, Waltham, MA, USA) was used to measure the concentration of human IL-6 released, according to manufacturer’s protocol. In the case of THP-1 cells, IL-6 was measured in cell lysate obtained at the end of each treatment using RIPA buffer and protein concentration was determined with Bradford assay.

### 2.12. Statistical Analysis

Data are shown as mean ± SD or frequency (%) of at least three independent biological replicates. A paired sample *t* test was used for RT-qPCR, ELISA and densitometry data analysis. Data analysis was performed using IBM SPSS Statistics for Windows, version 25 (IBM Corp, Armonk, NY, USA). Statistical significance was defined as a two-tailed *p*-value < 0.05.

## 3. Results

### 3.1. Chemical Characterisation of OLE

To determine the in vitro anti-inflammatory properties of OLE and its active compounds Oleacin (OC) and Oleuropein-Aglycone (OA), we first produced an aqueous OLE in PBS (Figure 1A) and characterised it in terms of total phenolic content (TPC). The TPC extraction yield resulting from five preparations (569.5 ± 22.142 µg/mL) is represented in Figure 1B. We also characterised OLE’s individual components and found that the most abundant were Oleuropein, Hydroxytyrosol, Oleacein and Oleuropein-aglycone. As expected, oleuropein was the most concentrated component, followed by Hydroxytyrosol. Oleacein and Oleuropein-aglycone were also present at high concentrations (Figure 1B). HRMS spectra, accurate masses, characteristic fragmentations, and retention times are shown in Supplementary Figure S1. Phenolic compounds are naturally bioactive molecules that show interesting antioxidant activity thanks to their hydroxylated aromatic rings. OC and OA structures are represented in Figure 1C.

### 3.2. Olive Leaf Extract, Oleacein and Oleorupein-Aglycone’s Effect on HUVEC, THP-1 and hSAEC Cell Viability

**Figure 2:**
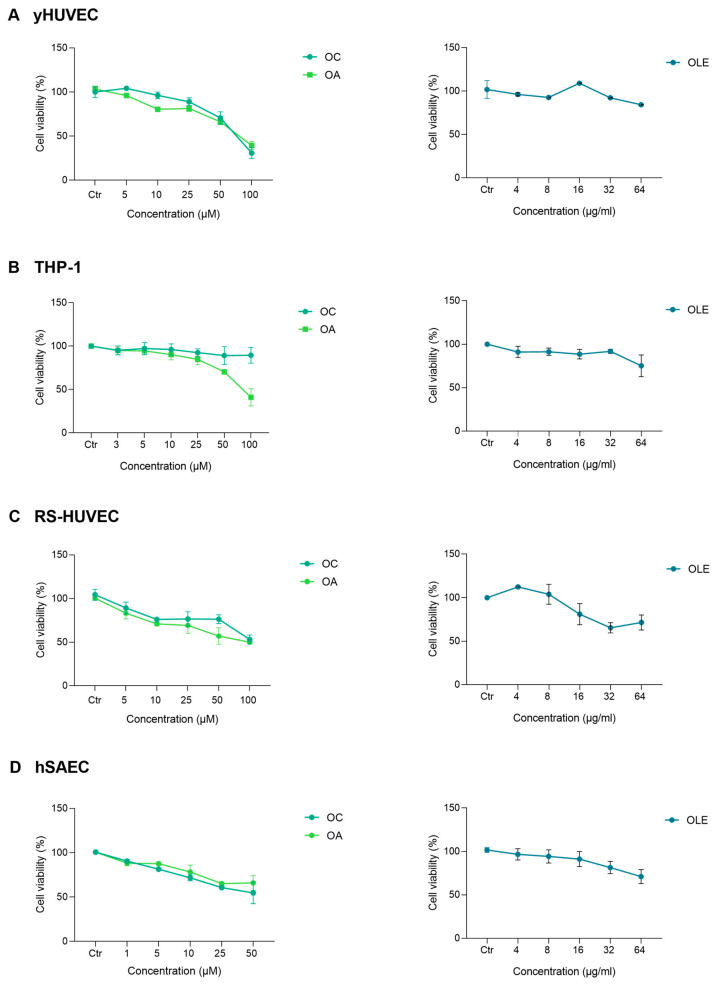
Effect of OLE, OC and OA on HUVEC, THP-1 and hSAEC cell viability. Cells were treated with OLE (from 4 to 64 μg/mL), OC and OA (from 1 to 100 μM) for 24 h. The results are expressed as a percentage of cell viability normalised to the viability of DMSO-treated cells (CTR) and represented as mean value ± SEM from three independent experiments. (**A**) yHUVECs. (**B**) RS-HUVECs. (**C**) THP-1. (**D**) hSAECs.

The cytotoxicity of OLE (different concentration of total TPC), oleacin (OC) or oleuropein-aglycone (OA) was assayed in young HUVEC (yHUVEC), RS-HUVEC, THP-1 and hSAEC cells after 24 h treatment by MTT assay (Figure 2). Subsequent experiments were performed considering the concentration of each compound and OLE that would ensure at least 85% of the viability of treated cells: yHUVECs, RS-HUVECs, THP-1 (5 μM OC and OA) and hSAEC (1 μM OC, 5 μM OA); for all cell types, 4 μg/mL OLE was used.

### 3.3. Anti-Inflammatory Activity of Olive Leaf Extract in LPS-Induced HUVECs and THP-1

In order to assess the anti-inflammatory properties of OLE and its active compounds, we first used LPS treatment to induce HUVEC and THP-1 activation. Both HUVECs and THP-1 were pre-treated with OLE, OC or OA for two hours and then treated with LPS for three hours. The inflammatory response was evaluated through IL-1β, Il-6, TNF-α and IL-8 cytokines and VCAM and ICAM-1 mRNA expression analyses. The release of IL-6 was also assayed in the conditioned medium.

**Figure 3:**
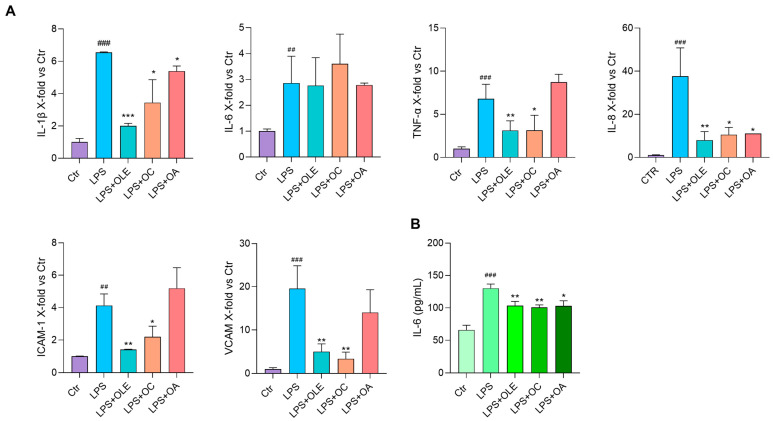
Anti-inflammatory activity of OLE, OC and OA in LPS-stimulated HUVEC cells. Cells were pre-treated with OLE (4 μg/mL), OC and OA (5 μM) for 2 h and stimulated with LPS (500 ng/mL) for 3 h. (**A**) IL-1β, IL-6, TNF-α, IL-8, ICAM-1 and VCAM mRNA expression analysis. Data were reported as fold change vs. untreated LPS-stimulated HUVEC (Ctr) according to 2^−ΔΔCt^ method and using β-actin as housekeeping. (**B**) IL-6 (pg/mL) in the culture medium; histograms represent the mean of three independent experiments ± SD. Paired *t* test, * *p* < 0.05, ** *p* < 0.01, *** *p* < 0.001 vs. HUVEC + LPS (LPS); ## *p* < 0.01, ### *p* < 0.001 vs. LPS-untreated HUVEC (Ctr).

In HUVECs, OLE significantly reduced the expression of all LPS-induced inflammatory markers; OC gave similar results and OA inhibited IL-1β and IL-8 mRNA expression (Figure 3A). Interestingly, whereas OLE, OC and OA’s effect on IL-6 mRNA expression was not appreciable, they exerted a significant and similar reduction in IL-6 secretion (Figure 3B, Table 1).

**Figure 4:**
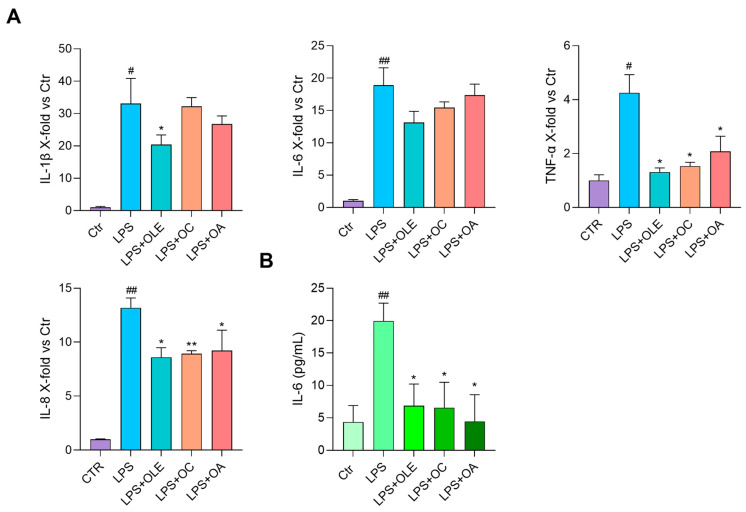
Anti-inflammatory activity of OLE, OC and OA in LPS-stimulated THP-1 cells. Cells were pre-treated with OLE (4 μg/mL), OC and OA (5 μM) for 2 h and stimulated with LPS (500 ng/mL) for 3 h. (**A**) IL-1β, IL-6, TNF-α and IL-8 mRNA expression. Data are reported as fold change vs. untreated LPS-stimulated THP-1 according to 2^−ΔΔCt^ method and using β-actin as housekeeping. (**B**) IL-6 (pg/mL) in cell lysates; histograms represent the mean of three independent experiments ± SD. Paired *t* test, * *p* < 0.05, ** *p* < 0.01 vs. THP-1 + LPS (LPS); # *p* < 0.05, ## *p* < 0.01 vs. LPS-untreated THP-1 (Ctr).

In THP-1, OLE again seemed to be efficient in inhibiting the expression of all markers tested, especially TNF-α, whose levels were reduced to those of unstimulated cells. OC and OA efficiently modulated almost all markers except for IL-1β and IL-6 mRNAs (Figure 4). Of note and again, OLE and its compounds significantly reduced the production of IL-6 that, in this case, was evaluated in the cell lysates (Figure 4B, Table 1), because its release in the conditioned medium was not appreciable, maybe due to the limited amount of time of the LPS treatment.

### 3.4. OLE, OC and OA Exert Anti-SASP Activity on RS-HUVECs

**Figure 5:**
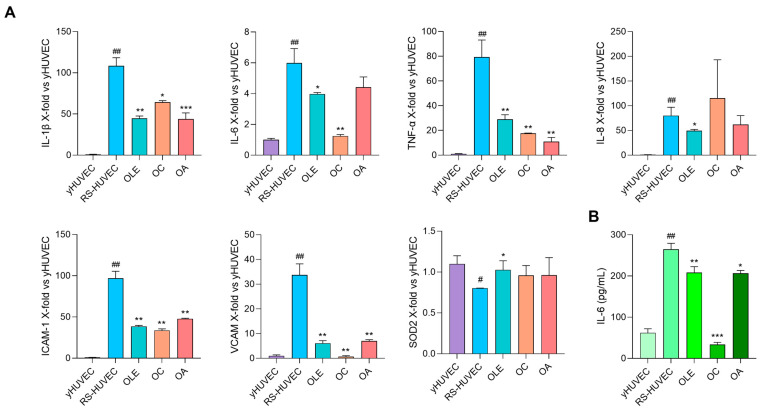
OLE, OC and OA exert anti-inflammatory activity on RS-HUVEC. (**A**) IL-1β, IL-6, TNF-α, IL-8, ICAM-1, VCAM and SOD2 mRNA expression in RS-HUVECs treated for 24 h with OLE (4 μg/mL), OC and OA (5 μM). Data are reported as fold change vs. Ctr (yHUVEC) according to 2^-ΔΔCt^ method, using β-actin as loading control. (**B**) IL-6 (pg/mL) in the culture medium; histograms represent the mean of three different experiments ± SD. Paired *t* test, * *p* < 0.05, ** *p* < 0.01, *** *p* < 0.001 vs. untreated RS-HUVEC, # *p* < 0.05, ## *p* < 0.01 vs. yHUVEC.

The effect of OLE, OC and OA was investigated in RS-HUVECs, a well-established model of cellular senescence, already characterised and described in our previous works for their SASP phenotype [24]. Figure 5 shows RS-HUVECs’ pro-inflammatory phenotype compared to yHUVECs. Treatments with OLE, OC or OA for 24 h significantly reduced the mRNA expression of all inflammatory markers tested (IL-1β, TNF-α, ICAM-1, VCAM) except for IL-8 which was modulated only by OLE (Figure 5A). Moreover, IL-6 release in the conditioned medium was efficiently reduced by all conditions (Figure 5B). Because replicative senescence is closely related to increased oxidative stress [37], we also decided to test the expression of SOD2. Results showed that it was significantly reduced in RS-HUVEC compared to yHUVEC and that treatment with OLE successfully restored its expression, with an effect almost comparable to yHUVEC; a slight, but not significant, increase with OC and OA was also observed (Figure 5A, Table 2).

### 3.5. OLE, OC and OA Have an Anti-Inflammatory and Anti-Viral Effect on hSAEC

**Figure 6:**
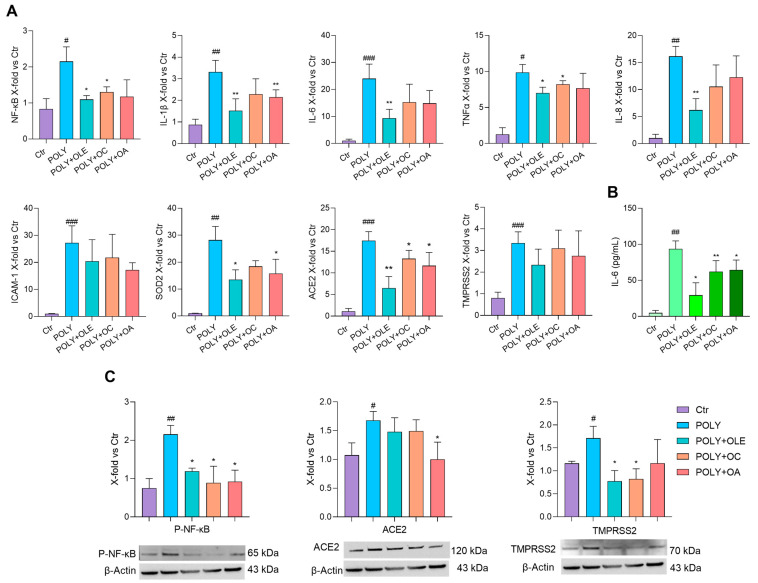
OLE, OC and OA anti-inflammatory and anti-viral effects on hSAEC cells. Cells were pre-treated with OLE (4 μg/mL), OC (1 μM) and OA (5 μM) for 2 h and then stimulated with Poly(I:C) for 24 h. (**A**) NF-kB, IL-1β, IL-6, TNFα, IL-8, ICAM-1, SOD2, ACE2 and TMPRSS2 mRNA expression. Data are shown as fold change vs. untreated Poly(I:C)-stimulated hSAEC according to 2^-ΔΔCt^ method, using actin as housekeeping. (**B**) IL-6 (pg/mL) in the culture medium. (**C**) Representative Western blot analysis showing P-NF-κB, ACE2 and TMPRSS2 protein expression, with β-actin as loading control. The results are expressed as mean ± SD from three independent biological replicates. Paired *t* test, # *p* < 0.05, ## *p* < 0.01, ### *p* < 0.001 vs. untreated hSAEC (CTR); * *p* < 0.05, ** *p* < 0.01 vs. Poly(I:C)-stimulated hSAEC (POLY).

Human small airway epithelial cells (hSAECs) were exploited as a robust in vitro model to study SARS-CoV-2 infection and replication, mainly for the presence of the key receptors angiotensin-converting enzyme 2 (ACE2) and transmembrane protease serine 2 (TMPRSS2) [38]. Preliminary tests by us and others indicated that LPS was inefficient in inducing inflammation in hSAEC cells [35,36] (Supplementary Figure S2). Hence, Polyinosinic:polycytidylic acid (Poly(I:C)), a double-stranded RNA similar to respiratory viruses such as influenza A virus and SARS-CoV-2 [36,39], was used to successfully stimulate hSAECs (Supplementary Figure S2B) and to evaluate the potential anti-inflammatory and anti-viral activity of OLE, OC and OA. Poly(I:C)-stimulated cells were pre-treated for 2 h with OLE (4 μg/mL), OC (1 μM) and OA (5 μM) and their effect on pro-inflammatory markers and furthermore on ACE2 and TMPRSS2 expression was analysed. Moreover, because SARS-CoV-2 is a potent NF-κB inducer [40], we decided to test the effect of OLE, OC and OA on NF-κB mRNA and P-NF-κB protein expression. VCAM was not upregulated upon preliminary experiments; therefore, it was not further analysed. Poly(I:C) successfully induced the mRNA expression of NF-kB, IL-1β, IL-6, TNF-α, IL-8 and ICAM-1 and almost all were inhibited significantly by OLE except for ICAM-1 (Figure 6A). OC and OA exerted their efficacy in lowering NF-κB and IL-1β, respectively. P-NF-kB protein expression reflected NF-kB mRNA modulation (Figure 6C). Notably, the release of IL-6 was significantly reduced by all the treatments (Figure 6B). Because SARS-CoV-2 infection showed more oxidative stress than other viral infections and considering that a correlation between increased oxidative stress and severe COVID-19 patients has been found [41], we also tested the expression of oxidative stress genes. Stimulation with Poly(I:C) induced SOD2 gene expression (Figure 6A), which was significantly reduced with OLE and OA, whereas we could not appreciate any modulation of SOD1 (Supplementary Figure S3). Concerning ACE2 and TMPRSS2, Poly(I:C) efficiently induced their mRNA expression and pre-treatments significantly reduced ACE2 mRNA but not TMPRSS2. Surprisingly, at the protein level, ACE2 was significantly downregulated only by OC, whereas TMPRSS2 was downregulated by OLE and OA (Figure 6C, Table 3).

## 4. Discussion

In recent years, olive tree by-products, such as olive leaves, have been deeply studied for their invaluable source of bioactive compounds useful as anti-inflammatory and antioxidant agents. The olive leaf total extract (OLE) and its derived compounds, mainly oleuropein and hydroxytyrosol, have been tested in several in vitro and in vivo systems showing similar properties to EVOO. They can reduce inflammation and oxidative species production in experimental models of gastric and intestinal diseases [7,8,16]; they also exert beneficial effects in metabolic syndrome, atherosclerosis and cardiovascular diseases by suppressing the inflammatory response, reducing lipid peroxidation and attenuating hypertension [11,13,14,42].

Accordingly, here we have demonstrated the valuable benefits of OLE and two of its less-studied components—OC and OA—that we directly purified from OLE using a cost-effective and environmentally sustainable method. Specifically, these molecules efficiently reduced the inflammatory response of (i) two of the cell types always involved in host responses to pathogens, i.e., monocyte/macrophages and endothelial cells (LPS-treated HUVECs and THP-1) [43], (ii) small airway epithelial cells, central to the pathogenesis of lung injury following environmental agent inhalation (hSAECs treated with Poly(I:C) to mimic a viral infection) and (iii) endothelial senescent cells (RS-HUVECs), characterised by a SASP phenotype which negatively affects the dynamics of the host response to pathogens [44]. Indeed, even if the beneficial effects of OLE on the endothelium have been greatly investigated [11,13,42], the modulatory effect of OC and OA on endothelial cell activation due to severe infection has been little explored. Of note, our results showed that in both LPS-stimulated HUVECs and THP-1, pre-treatment with OLE, OC and to a lesser extent with OA was effective in reducing the mRNA expression of most of the cytokines and in the case of endothelial cells all the adhesion molecules tested. Notably, all treatments still successfully reduced IL-8 mRNA and IL-6 release in the conditioned medium. Due to the pivotal role played by these cytokines in LPS-induced inflammatory response, these data highlight the efficacy of both OC and OA as anti-inflammatory compounds even if OLE, which indeed contains a mix of more active compounds is the most effective.

Based on these data, we were encouraged to analyse the effects of the above-mentioned extract and compounds in endothelial senescent cells, an in vitro model of chronic inflammation. It has been now widely recognised that aging is associated with progressive cellular senescence and dysfunction. One of the main features of senescent cells is the SASP phenotype, which exerts a detrimental paracrine effect within the tissues (even in the lung [45]) on one hand and contributes to inflammaging on the other one [27], thus playing a role in the development of Age-Related Diseases (ARDs). In this framework, we demonstrated for the first time that OLE and its compounds can inhibit the synthesis and the release of pro-inflammatory cytokines characterising the SASP phenotype and furthermore the expression of adhesion molecules. Therefore, it can be assumed that they could be useful to reduce leucocyte recruitment, progressive tissue dysfunction and also systemic inflammation in vivo during aging (Figure 5). Moreover, our results showed that OLE can restore RS-HUVECs’ antioxidant activity, by upregulating SOD2 mRNA expression (Figure 5A), an enzyme that prevents the age-dependent endothelial cell dysfunction and apoptosis by serving as a first line of defence against superoxide anion radical toxicity [46]. The possibility of using OLE and its components to control both endothelial dysfunction and oxidative stress could represent valid help to promote what has been called the Health Aging process [47].

These data are also important in light of the recent pandemic of COVID-19 that showed that inflammaging could be relevant for severe lung viral infections pathogenesis and progression because the case fatality rate grows exponentially with age and comorbidities [48]. Indeed, SARS-CoV-2 infection can cause systemic hyperinflammation [49] accompanied by the so-called “cytokine storm” and possibly the subsequent multi-organ failure. In this context, we wanted also to investigate the potential role of OLE and its bioactive compounds in modulating small airway epithelial cells’ activation upon viral infection. We exploited Poly(I:C)-treated hSAEC cells, which outline a model system that reliably mirrors the physiology and architecture of the lung epithelium, thus representing an effective tool to study the molecular mechanisms underlying respiratory viral infections, such as SARS-CoV-2 [50,51]. Our findings mostly agreed with the other models of bacterial-induced acute inflammation, because OLE was able to reduce the synthesis and release of all the pro-inflammatory cytokines and markers tested; concerning OC and OA, we observed for the first time that they inhibited NF-kB, TNF-α and IL-1β mRNA expression, respectively, and both IL-6 release and NF-kB phosphorylation (Figure 6). The radical scavenging activity of OLE has long been known [52]. Here, we showed that OLE and OA reduced the transcription of SOD2, which was upregulated upon Poly(I:C)-stimulation as an NF-kB responsive gene [53] (Figure 6), highlighting the contribution of these compounds in restoring the impaired redox homeostasis in the context of acute viral infections [41]. On the contrary, we could not appreciate SOD1 modulation, in line with the literature [54] (Supplementary Figure S3).

Finally, because several in silico computational studies reported an efficient molecular interaction between olive leaf metabolites and SARS-CoV-2 main viral targets [55], we wanted to test the antiviral activity of OC and OA, by analysing their ability to counteract the increasing expression of SARS-CoV-2 receptor ACE2 for entry and the serine protease TMPRSS2 for S protein priming upon Poly(I:C) stimulation [56,57]. Our results showed opposite trends between ACE2 and TMPRSS2 at mRNA or protein expression levels: OLE, OC and OA were effective in reducing ACE2 mRNA expression, whereas they could not significantly reduce TMPRSS2; on the contrary, OLE and OC were efficient in decreasing TMPRSS2 protein expression, whereas only OA could lower ACE2 (Figure 6). The intricate role of ACE2 in the pathophysiology of respiratory viral infections is yet to be unravelled. It has been demonstrated that ACE2 and TMPRSS2 are differentially expressed depending on genetics, age and comorbidities, with significant upregulation in the elderly, smokers and COPD patients [41,57]. Therefore, the reduction in ACE2 mRNA expression levels after OLE, OC and OA treatment could be explained as a positive effect to counteract an inflammatory status; however, because ACE2 also exerts a protective effect in the lungs, by targeting angiotensin II [58], we can hypothesise the existence of a post-transcriptional regulatory mechanism that ensures a basal level of ACE2 protein expression in our cellular system. On the other hand, it has been shown that TMPRSS2-mediated ACE2 cleavage is even more harmful to the host and that an increased expression of TMPRSS2—due to genetics, age or comorbidities—could exacerbate the course of COVID-19 [59]. Our findings showed that although OLE, OC and OA were not effective in downregulating TMPRSS2 mRNA expression (Figure 6A), OLE and OC could successfully modulate the protein expression (Figure 6C). Therefore, we can assume that using OLE or its bioactive compounds could ameliorate infection prognosis.

## 5. Conclusions

Overall, this work strengthens the already existing data on the beneficial effects of olive leaf extract intake as an anti-oxidant, anti-bacterial and anti-inflammatory agent [14,60,61,62], also in upper respiratory illnesses [63]. In addition, it suggests its use to modulate the cytokine storm occurring during several forms of viral infections, especially in aged individuals. Our data also invite researchers (i) to deeply investigate the role and (ii) consider the eventual use of the two less-studied active OLE derivatives, oleacin and oleuropein-aglycone, in controlling inflammation. Their production from OLE is simple, safe, cost-effective and with a low environmental impact. OC and OA could, therefore, be formulated for oral administration in case of systemic inflammation, i.e., inflammaging, and infectious diseases. Moreover a specific formulation for aerosol intake to reduce or prevent local airway infection can be also hypothesised.

In fact, these bioactive compounds as a supplement may reduce the doses or the time of administration of conventional drugs, while maintaining their efficacy. However, while oral use of OLE has already been tested in several human clinical trials and commercial supplements are available, in vivo studies for OC and OA are still very few in humans and also in mice [64,65]. Hence, further studies are needed to test their toxicity and the best formulation for their administration. OC and OA bioactive and mucoadhesive formulations for systemic and local treatments are currently under investigation in our laboratory.

## Figures and Tables

**Table 1:** Effect of OLE, OC and OA in LPS-treated yHUVECs (**A**) and THP-1 (**B**).

A							
yHUVEC	IL-1β	IL-6	TNF-α	IL-8	ICAM-1	VCAM	IL-6 (pg/mL)
OLE	↓↓↓	-	↓↓	↓↓	↓↓	↓↓	↓↓
OC	↓	-	↓	↓	↓	↓↓	↓↓
OA	↓	-	-	↓	-	-	↓
**B**							
**THP-1**	**IL-1β**	**IL-6**	**TNF-α**	**IL-8**			**IL-6 (pg/mL)**
OLE	↓	-	↓	↓			↓
OC	-	-	↓	↓↓			↓
OA	-	-	↓	↓			↓

“↓” indicates statistically significant difference vs. LPS-treated controls. “↓” *p* < 0.05, “↓↓” *p* < 0.01, “↓↓↓” *p* < 0.001, “-” not significant.

**Table 2:** Effect of OLE, OC and OA in RS-HUVECs.

RS-HUVEC.	IL-1β	IL-6	TNF-α	IL-8	ICAM-1	VCAM	SOD2	IL-6 (pg/mL)
**OLE**	↓↓	↓	↓↓	↓	↓↓	↓↓	↓	↓↓
**OC**	↓	↓↓	↓↓	-	↓↓	↓↓	-	↓↓↓
**OA**	↓↓↓	-	↓↓	-	↓↓	↓↓	-	↓

“↓” indicates statistically significant difference vs. RS-HUVEC controls. “↓” *p* < 0.05, “↓↓” *p* < 0.01, “↓↓↓” *p* < 0.001, “-“ not significant.

**Table 3:** Effect of OLE, OC and OA in hSAECs.

mRNA									
hSAEC	NF-kB	IL-1β	IL-6	TNF-α	IL-8	ICAM-1	SOD2	ACE2	TMPRSS2
**OLE**	↓	↓↓	↓↓	↓	↓↓	-	↓	↓↓	-
**OC**	↓	-	-	↓	-	-	-	↓	-
**OA**	-	↓↓	-	-	-	-	↓	↓	-
**Protein**									
**hSAEC**	**IL-6 (pg/mL)**	**P-NF-kB** **(Protein)**		**ACE2** **(Protein)**	**TMPRSS2** **(Protein)**				
**OLE**	↓	↓		-	↓				
**OC**	↓↓	↓		-	↓				
**OA**	↓	↓		↓	-				

“↓” indicates statistically significant difference vs. Poly(I:C)-treated controls. “↓” *p* < 0.05, “↓↓” *p* < 0.01, “-“ not significant.

## Data Availability

Data are contained within the article and Supplementary material.

## Supplementary Materials

The following supporting information can be downloaded at https://www.mdpi.com/article/10.3390/antiox12081509/s1, Figure S1: UHPLC-UV-ESI-HRMS analysis. Figure S2: hSAEC-induced inflammation. Figure S3: SOD1 mRNA expression.
