# Supplementary material for: Anti-Inflammatory Effects of Olive Leaf Extract and Its Bioactive Compounds Oleacin and Oleuropein-Aglycone on Senescent Endothelial and Small Airway Epithelial Cells
Source: Antioxidants (Basel). 2023 Jul 28;12(8):1509. doi: 10.3390/antiox12081509 (PMC10451521; doi:10.3390/antiox12081509)
Supplement: Supplementary file 1 [file antioxidants-12-01509-s001.zip › antioxidants-2489049-supplementary.pdf]

antioxidants

MDPI

A

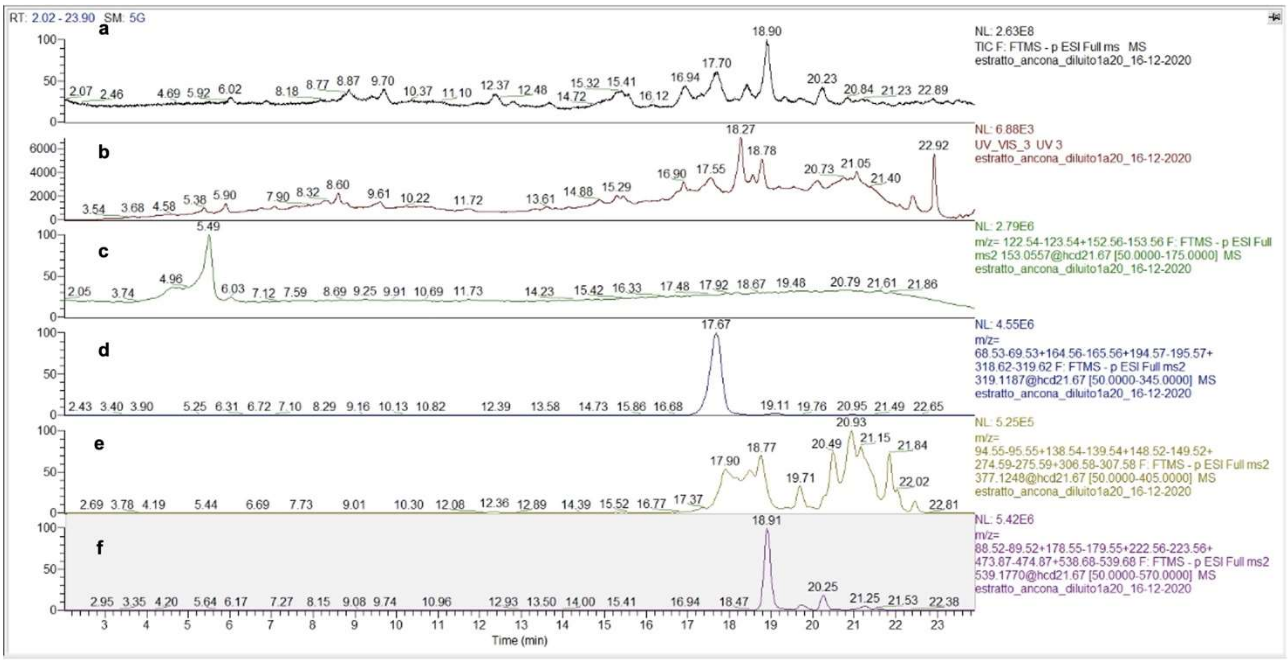

B

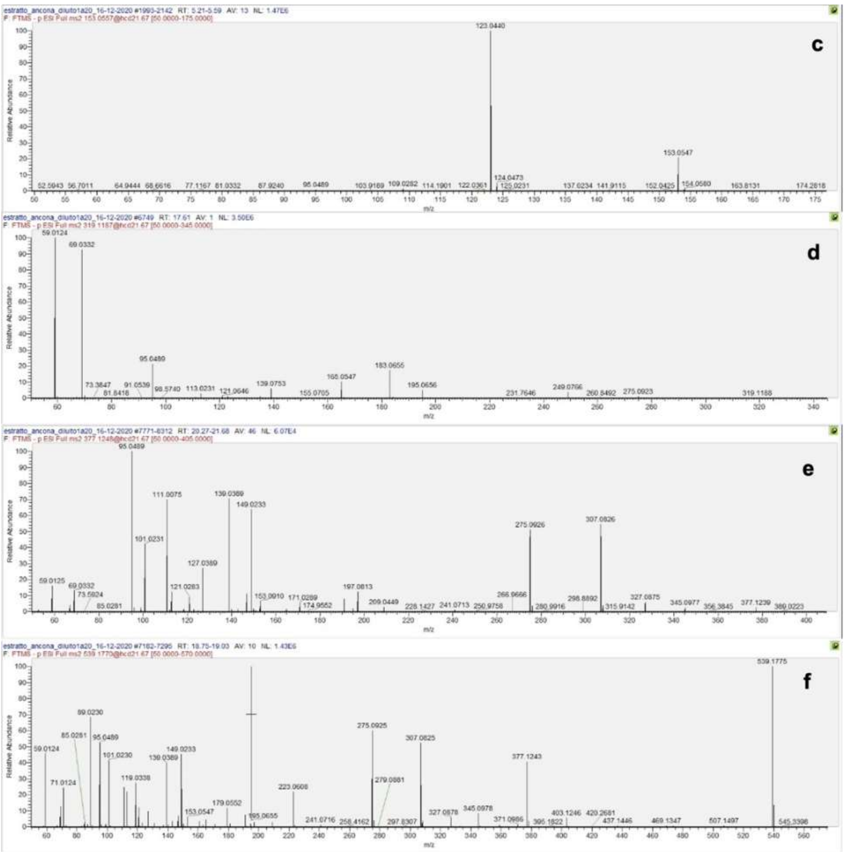

Figure S1. UHPLC-UV-ESI-HRMS analysis. (**A**) (a) LC-HRMS full scan chromatogram, (b) LC-UV chromatogram (280nm), and negative LC-HRMS-PRM (c) Hydroxytyrosol (153.0557 m/z), (d) Oleacin (319.1187 m/z), (e) Oleuropein aglycon (377.1248 m/z) and (f) Oleuropein (539.1770m/z) chromatograms. (**B**) HR-MSMS spectra of (c) Hydroxytyrosol (153.0557 m/z), (d) Oleacin (319.1187 m/z), (e) Oleuropein aglycon (377.1248 m/z) and (f) Oleuropein (539.1770 m/z).

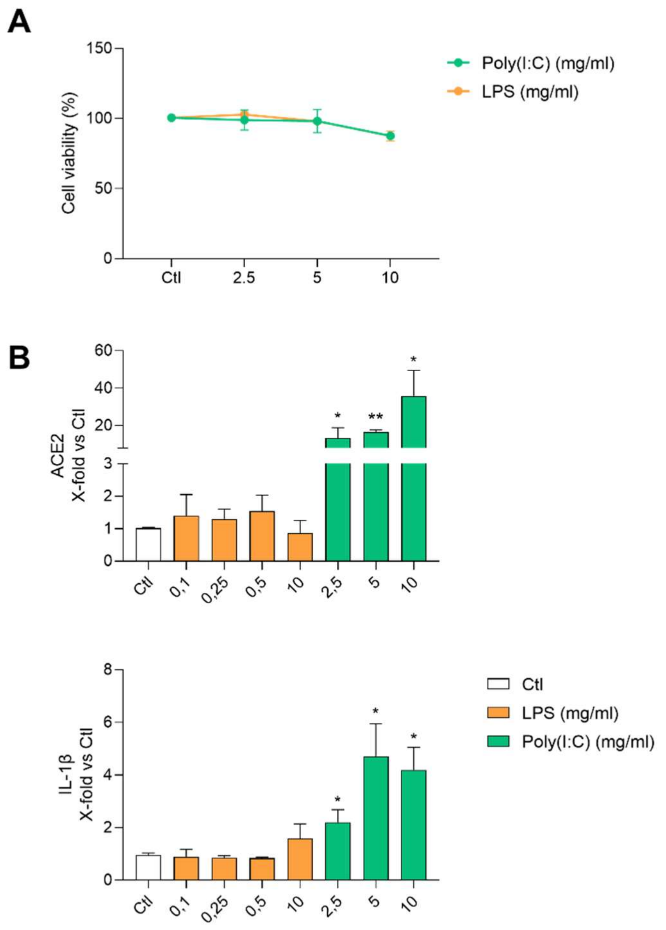

Figure S2. hSAEC-induced inflammation. (**A**) Dose-response curve of hSAEC to LPS and Poly(I:C). hSAEC were treated with different concentrations of LPS and Poly(I:C) (from 2.5 µM to 10 µM) or with DMSO alone as a control for 24h. Cell viability was determined by MTT assay. Results are expressed as a percentage of cell viability normalized to viability of DMSO-treated cells (Ctl) and presented as mean value ±SD from three independent biological experiments. (**B**) ACE2 and IL-1β mRNA expression in hSAEC cells treated with different concentrations of LPS and Poly(I:C). Data are shown as fold change vs untreated hSAEC cells (Ctr).

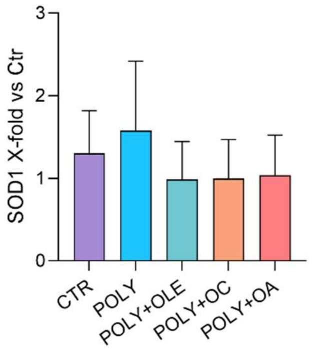

Figure S3. SOD1 mRNA expression. SOD1 mRNA expression in Poly(I:C)-stimulated hSAEC, treated with OLE, OC and OA for 24h. Data are shown as fold change vs untreated Poly(I:C)-stimulated hSAEC according to $2^{-\Delta\Delta Ct}$ method, using actin as housekeeping.
